# Low Subicular Volume as an Indicator of Dementia-Risk Susceptibility in Old Age

**DOI:** 10.3389/fnagi.2022.811146

**Published:** 2022-03-03

**Authors:** Sonja M. Kagerer, Clemens Schroeder, Jiri M. G. van Bergen, Simon J. Schreiner, Rafael Meyer, Stefanie C. Steininger, Laetitia Vionnet, Anton F. Gietl, Valerie Treyer, Alfred Buck, Klaas P. Pruessmann, Christoph Hock, Paul G. Unschuld

**Affiliations:** ^1^Institute for Regenerative Medicine, University of Zurich, Zurich, Switzerland; ^2^Psychogeriatric Medicine, Psychiatric University Hospital Zurich, University of Zurich, Zurich, Switzerland; ^3^Institute for Biomedical Engineering, University of Zurich and ETH Zürich, Zurich, Switzerland; ^4^Department of Nuclear Medicine, University Hospital Zurich, University of Zurich, Zurich, Switzerland; ^5^Neurimmune, Schlieren, Switzerland; ^6^Geriatric Psychiatry, Department of Psychiatry, University Hospitals of Geneva, University of Geneva, Geneva, Switzerland

**Keywords:** ultra-high field MRI, 7 Tesla, hippocampus subfield segmentation, prodromal AD, episodic memory, iron, subiculum, Alzheimer’s disease

## Abstract

**Introduction:**

Hippocampal atrophy is an established Alzheimer’s Disease (AD) biomarker. Volume loss in specific subregions as measurable with ultra-high field magnetic resonance imaging (MRI) may reflect earliest pathological alterations.

**Methods:**

Data from positron emission tomography (PET) for estimation of cortical amyloid β (Aβ) and high-resolution 7 Tesla T1 MRI for assessment of hippocampal subfield volumes were analyzed in 61 non-demented elderly individuals who were divided into risk-categories as defined by high levels of cortical Aβ and low performance in standardized episodic memory tasks.

**Results:**

High cortical Aβ and low episodic memory interactively predicted subicular volume [*F*(3,57) = 5.90, *p* = 0.018]. The combination of high cortical Aβ and low episodic memory was associated with significantly lower subicular volumes, when compared to participants with high episodic memory (*p* = 0.004).

**Discussion:**

Our results suggest that low subicular volume is linked to established indicators of AD risk, such as increased cortical Aβ and low episodic memory. Our data support subicular volume as a marker of dementia-risk susceptibility in old-aged non-demented persons.

## Introduction

Cortical deposition of amyloid β (Aβ) plaques is a hallmark of Alzheimer’s Disease (AD), which is clinically characterized by deficits in episodic memory performance as the most typical and earliest clinical feature (Braak and Braak, 1991; Scheltens et al., 2016). The formation of Aβ plaques is believed to be one of the earliest changes in the hypothesized pathophysiological continuum of AD and to start decades before manifestation of the clinical syndrome (Braak and Braak, 1997; Jack et al., 2010; Sperling et al., 2011; Villemagne et al., 2013; Jansen et al., 2015; Parker et al., 2019). Several studies on healthy old-aged persons have consistently demonstrated an association between increased brain Aβ burden, reduced hippocampal volume, and increased risk for cognitive dysfunction (Rentz et al., 2010; Rodrigue et al., 2012; Vos et al., 2013; Hsu et al., 2015; Donohue et al., 2017; Bilgel et al., 2018; Fletcher et al., 2018; Haller et al., 2019; Parker et al., 2019; Svenningsson et al., 2019; Yassi et al., 2019). While the precise mechanism of how years of Aβ accumulation in the human brain may be linked to dementia is still not very well-understood, increased cerebral Aβ plaque load is nevertheless considered to represent a major risk factor for developing AD at old age (Tomlinson et al., 1968; Knopman et al., 2003; Bennett et al., 2006; Mintun et al., 2006; Rowe et al., 2007; Marchant et al., 2012; Jack et al., 2014). It is included in the criteria for the preclinical stages of AD and is increasingly used in clinical trials (Sperling et al., 2011; Sevigny et al., 2016). Yet, the predictive value of increased Aβ burden is compromised by a lack of specificity (Tomlinson et al., 1968; Mintun et al., 2006; Jack et al., 2014), and susceptibility of an individual at risk to eventually develop AD is known to be moderated by several factors (Prestia et al., 2015). Interestingly, risk for pathological cognitive decline at old age is significantly elevated when increased cerebral Aβ plaque load is associated with an already subtle cognitive dysfunction (Insel et al., 2016). Hence, further characterization of brain phenotypes (such as morphology) may contribute to a better understanding of the relationship between high cerebral Aβ plaque load in cognitively unimpaired subjects, and their individual risk for cognitive decline.

Subtle changes in episodic memory performance within the normal range may represent a prequel of the gradual progression of cognitive dysfunction in pre-clinical AD (Linn et al., 1995; Lange et al., 2002; Tierney et al., 2005; Albert, 2011; Bastin and Salmon, 2014; Pietrzak et al., 2015). Episodic memory dysfunction in old-aged persons is closely linked to hippocampal volume loss (Jack et al., 2000; Barnes et al., 2009; Dickerson and Eichenbaum, 2010; Gorbach et al., 2017; Nyberg, 2017). Considering a high degree of assumed functional specialization, atrophy of distinct hippocampal subfields may even better reflect earliest pathological changes of AD trajectories (de Flores et al., 2015a; Perrotin et al., 2015; Lindberg et al., 2017; Evans et al., 2018; Liang et al., 2018; Madusanka et al., 2019; Parker et al., 2019; Zhao et al., 2019; Dounavi et al., 2020; McKeever et al., 2020; Wong et al., 2020; Zhang et al., 2020).

Based on these earlier reports, we hypothesized that volume of distinct hippocampal subfields of cognitively normal old-aged adults may be associated with an individual’s risk-level for developing AD, as reflected by cortical Aβ-plaque deposition and episodic memory performance.

## Materials and Methods

### Participants

Participants were recruited in the cantone of Zurich, Switzerland, from ongoing studies at our center. All studies were conducted in compliance with local regulatory requirements, the Declaration of Helsinki and approved by the cantonal ethics committee of Zurich, Switzerland (World_Medical_Association., 1991).

Inclusion criteria for this project were age equal or above 50 years, written informed consent as approved by the local ethics authority, German language proficiency and unimpaired overall cognitive status as indicated by Mini−Mental State Examination (MMSE) ≥ 27/30. Exclusion criteria were presence of any condition possibly affecting cognition or study participation (e.g., severe hearing loss), any current medication or substance abuse with prompt effects on cognition, serious medical or psychiatric illness, any evidence of infarction or inflammation in the cranial MRI, contraindications to MRI or PET, or significant exposure to radiation. A total of 69 non-demented, cognitively normal old-aged persons completed both PET for brain Aβ-plaque burden (11C-PiB- or 18F-flutemetamol-PET) and high-field MRI at 7 T. Neuropsychological data was obtained for 68 participants. When stratifying the study sample by median split, seven participants with episodic memory or SUVR composite score equal to the median, were excluded.

### Cognitive Assessment of Participants

All persons included in this analysis received standardized neuropsychological testing. Screening for cognitive impairment was performed by applying the MMSE (Folstein et al., 1975) and the Consortium to Establish a Registry for Alzheimer’s Disease (CERAD) neuropsychological battery (Sotaniemi et al., 2012). Episodic memory performance was assessed with the German Verbal Learning and Memory Test (VLMT) (Feurle et al., 1990; Helmstaedter et al., 2001), which is an adapted German language version of the Rey Auditory Verbal Learning Test (AVLT) (Muller et al., 1997; Lezak, 2004). The VLMT includes serial learning and recall of a 15-item word list over five trials. Recall performance was assessed immediately after reading the word list to the participant and after distraction (VLMT delayed recall measure). We created a composite measure of episodic memory by combining the z-standardized scores for immediate and delayed recall of the VLMT results for a more comprehensive assessment of episodic memory performance (Bastin and Salmon, 2014; Jonaitis et al., 2019).

### Magnetic Resonance Imaging Acquisition, Pre-processing, and Estimation of Hippocampal Subfield Volumes

All subjects were scanned using a Philips 7T Achieva whole-body scanner (Philips Healthcare, Best, Netherlands) equipped with a Nova Medical quadrature transmit head coil and 32-channel receive coil array, located at the Institute for Biomedical Engineering (IBT) at the Swiss Federal Institute of Technology at Zurich, Switzerland (ETH Zurich). A high-resolution T1-weighted 3D MP2RAGE image (TR/TE = 4.8 ms/2.1 ms, voxel size = 0.6 mm × 0.6 mm × 0.6 mm, SENSE-factor = 2 × 1 × 2, scan duration = 7:50 min) was acquired for anatomical referencing of brain structures and automated image segmentation.

Preprocessing of the T1 images was performed using the FreeSurfer software package Version 6.0 (Dale et al., 1999; Fischl et al., 1999, 2002, 2004a,b; Fischl and Dale, 2000; Han et al., 2006; Jovicich et al., 2006; Reuter et al., 2012). Pre-processing involved conversion from the three-dimensional nifti-format, motion correction and average of multiple volumetric T1-weighted images, transformation to Talairach space, intensity normalization, brain extraction, segmentation of the subcortical white and deep gray matter volumetric structures, tessellation of the gray and white matter boundary, automated topology correction, and surface deformation to optimally place the gray/white and gray/cerebrospinal fluid boundaries.

Hippocampal subfield segmentation was performed using algorithms implemented in FreeSurfer Version 6.0^1^ (Iglesias et al., 2015). Bilateral hippocampi were segmented into 12 subfields each, representing across hemisphere averages as no lateralized hypothesis was followed: parasubiculum, presubiculum, subiculum, cornu ammonis (CA)1, CA2/3, CA4, granule cell layer of dentate gyrus (GC-DG), hippocampus-amygdala-transition-area (HATA), fimbria, molecular layer, hippocampal fissure, and hippocampal tail. Hippocampal segmentation quality was manually controlled for every participant. Intracranial volume (ICV), including brain tissues and other biological materials such as meninges and cerebrospinal fluid, was also estimated by applying FreeSurfer algorithms (Fischl and Dale, 2000).

### Pittsburgh Compound-B-Positron Emission Tomography

Estimation of individual brain Aβ load was performed in 30 participants by applying 11C-labeled Pittsburgh Compound-B (PiB) positron emission tomography (PET) (Mathis et al., 2003; Klunk et al., 2004; Solbach et al., 2005). 11C-PiB PET scans were performed at the PET Center of the Division of Nuclear Medicine, Zurich University Hospital, as reported earlier by our group (Steininger et al., 2014; Gietl et al., 2015). In brief, an individual dose of approximately 350 MBq of 11C-labeled PiB was applied using intravenous access to the cubital vein. Images were corrected for attenuation (low-dose CT-based) and standard quantitative filtered back projection algorithm including necessary corrections for scatter, randoms and dead time were applied.

Cerebral amyloid deposition values were extracted from late-frame signals (minutes 50–70) using a standard routine as implemented in PMOD Brain Tool software-package (PNEURO, Version 3.4, PMOD Technologies Ltd., Zurich, Switzerland). As a single measure of individual cortical Aβ load, cortical PiB SUVR were calculated as reported earlier (Steininger et al., 2014; Gietl et al., 2015). In brief, a composite score based on cortical gray matter segmented regions of interest defined from Hammers maximum probability atlas (as implemented in PMOD Neuro Tool) were averaged and divided by cerebellar gray matter PIB Values. Regions of occipital lobe, insula and primary motor and sensorimotor were excluded. Gray matter segmentation as well as regions of interest analysis was performed on individual 3D MRI scans acquired close to the PET visit and visually controlled. We defined a PiB-measured “amyloid-positivity” by a mean cortical PiB-SUVR threshold of 1.4, as described earlier (Roberts et al., 2017).

### Flutemetamol-Positron Emission Tomography

Thirty one subjects were recruited from another ongoing study at our center, which used 18F-flutemetamol-PET to estimate individual cortical brain Aβ-plaque-load as described earlier by our group (van Bergen et al., 2018). The 18F-flutemetamol-PET was conducted at our research facilities at the Institute for Regenerative Medicine in Schlieren (IREM), Switzerland. An individual dose of 140 MBq of 18F-flutemetamol was injected into the cubital vein. Time-of-flight algorithms including necessary corrections were applied to reconstruct late frame PET-images (minutes 85–105). Standard MR imaging-based attenuation correction images were used to derive attenuation correction maps, which were generated using standard procedures implemented by the manufacturer. The resulting 3D-volumes of flutemetamol uptake (matrix = 256 × 256 × 89, voxel size = 1.2 mm × 1.2 mm × 2.78 mm) were also processed with PMOD Neuro Tool as the 11C-PiB-PET volumes. For defining 18F-flutemetamol measured “amyloid-positivity,” a SUVR threshold of 1.56 was used, which has been defined in earlier 18F-flutematamol studies on AD and healthy controls (Vandenberghe et al., 2010).

### Estimation of Brain Amyloid β-Plaque- Density

To be able to merge Aβ-plaque-load measures from the 11C-PiB-PET and 18F-flutemetamol-PET-groups, 18F- flutemetamol-, and 11C-PiB-based SUVRs were normalized with their respective established positivity thresholds (1.56 for 18F-flutemetamol and 1.4 for PiB). The yielded ratios of individual Aβ-plaque-load versus the positivity cut-off score of all subjects were then merged. The thus obtained individual Aβ-plaque-load ratios were then used for stratification of the study population.

### Definition of Four Alzheimer’s Disease-Risk Groups and Statistical Analysis

A median split was performed on the episodic memory composite measure and the individual composite amyloid SUVR measure to divide the sample into four groups that reflect assumed individual risk for developing AD, as defined by earlier biomarkers studies on cognitive performance and brain Aβ-burden in old-aged, non-demented persons (Albert et al., 2001, 2011; Gomar et al., 2011; Jansen et al., 2015; Prestia et al., 2015; Elman et al., 2020; Papp et al., 2020). The four risk groups were defined as follows: (1) low-Aβ-high-episodic memory (lowest risk assumed), (2) low-Aβ-low-episodic memory, (3) high-Aβ-high-episodic memory, and (4) high-Aβ-low-episodic memory (highest risk assumed). To this effect, VLMT scores above the group median were categorized as “high episodic memory performance,” VLMT scores below the median as “low episodic memory performance.” Accordingly, composite SUVR scores above the group median of this study were categorized as “high-Aβ,” SUVR scores below the median as “low Aβ.”

Bilateral hippocampal subfield volumes were submitted to statistical analysis. Statistical analysis of volumes was designed to not include outliers, as defined by values of whole brain volume deviating more than 2.5 SD from the mean as this might point to a different pathology.

To test the effects of cortical Aβ and episodic memory on hippocampal subfield volumes, a multiple analysis of variance (MANOVA) was calculated with all 12 subfield volumes as dependent variables and group as independent variable. Following MANOVA, the group means of hippocampal subfield volumes were used for a cluster analysis (Mahalanobis distances) to identify AD risk subgroups with particular relevance for hippocampal subfield volumes. Mahalanobis distances is an established method to calculate statistic distances (independence) between data points (subgroups) (Mahalanobis, 1936; Kim, 2000; McLachlan, 2004). Statistic independence of data points is visualized in the resulting matrix of a hierarchichal binary clustertree (dendrogram), the graphical output of this function. Based on the results of this cluster analysis, the sample was then divided into a high-risk group with high cortical Aβ and low episodic memory performance and a low-risk group encompassing all other individuals. The ensuing one-way analyses of variance (ANOVA)s were calculated with all 12 subfield volumes as dependent variables and risk groups as independent variable. *Post hoc* analyses for each subfield were performed to compare their volumes between the high and the low-risk group and to test the interaction between cortical Aβ and episodic memory.

Multiple testing bias (false discovery rate, FDR) was allowed for using the Benjamini–Hochberg procedure (Benjamini and Hochberg, 1995). To this effect, all obtained *p*-values were FDR corrected, allowing estimation of test-significance at adjusted alpha = 5%.

## Results

### Sample Characteristics

The current analysis includes 61 cognitively normal old-aged study participants (Age 58–82 years; 22 females; Table 1).

**TABLE 1:** Demographic and other descriptive data.

	M(SD)
*N* (females/males)	61(22/39)
Age	70.23 (6.52)
Education	15.18 (2.97)
MMSE	29.16 (1.08)
VLMT—Immediate recall	9.59 (3.88)
VLMT—Delayed recall	9.18 (4.19)
Cortical 11C-PiB *n* = 31	1.24 (0.25)
Cortical 18f-flutemetamol *n* = 30	1.20 (0.32)
Hippocampal tail (mm^3^)	463.53 (72.89)
Subiculum (mm^3^)	364.82 (53.10)
CA1 (mm^3^)	549.99 (71.67)
Hippocampal fissure (mm^3^)	167.64 (29.71)
Presubiculum (mm^3^)	252.87 (38.75)
Parasubiculum (mm^3^)	55.22 (9.66)
Molecular layer (mm^3^)	475.84 (63.54)
GCMLDG (mm^3^)	247.28 (35.01)
CA3 (mm^3^)	180.40 (31.13)
CA4 (mm^3^)	215.40 (30.75)
Fimbria (mm^3^)	59.37 (18.25)
HATA (mm^3^)	52.17 (8.74)
eTIV (cm^3^)	1413.64 (209.55)

*CA, Cornu Ammonis; GC-DG, granule cell layer of the dentate gyrus; eTIV, estimated total intracranial volume; HATA, hippocampus-amygdala-transition-area; VLMT, Verbal Learning and Memory Test.*

Mean SUVR in study participants who received 11C-PiB-PET (*n* = 30) was 1.24 (SD = 0.25). In the group of study participants, who received 18F-flutemetamol-PET (*n* = 31), mean SUVR was 1.20 (SD = 0.32). Overall, 10 individuals were amyloid positive as defined by the established SUVR of ≥1.4 for PiB- and ≥1.56 for 18F-Flutemetamol-PET.

The average VLMT scores of all subjects were 9.59 (SD = 3.88) for the immediate recall and 9.18 (4.19) for the delayed recall.

Division of the sample by median split according to Aβ plaque load or episodic memory performance resulted in 30 subjects with low Aβ versus 31 subjects with high Aβ and 28 subjects with low episodic memory versus 33 subjects with high episodic memory. Allocation of the subjects to four groups representing AD risk generated group sizes of 13 subjects with high-Aβ-low-episodic memory, 17 subjects with high-Aβ-high-episodic memory, 15 subjects with low-Aβ-low-episodic memory, and 16 subjects with low-Aβ-high-episodic memory.

### Hippocampal Subfield Segmentation

**FIGURE 1:**
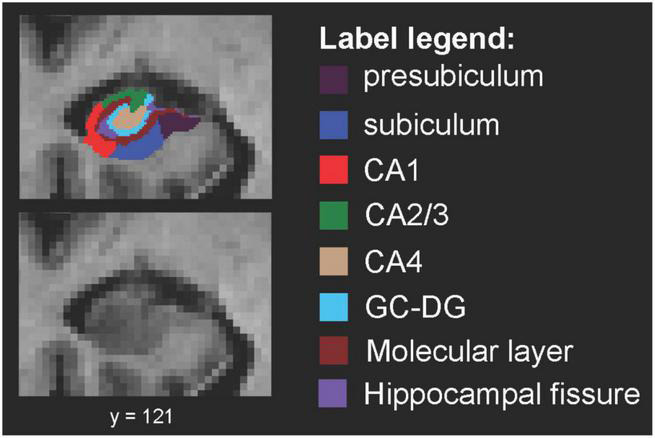
Spatial definition of hippocampal subfields by 7 Tesla MP2RAGE. Coronal view of right-hemispheric hippocampal subfield segmentation (radiological convention) from one example subject, defined by using FreeSurfer V6.0 for segmentation of high resolution 7 Tesla MP2RAGE volumes. CA, cornu ammonis; GC-DG, granule cell layer of the dentate gyrus.

**FIGURE 2:**
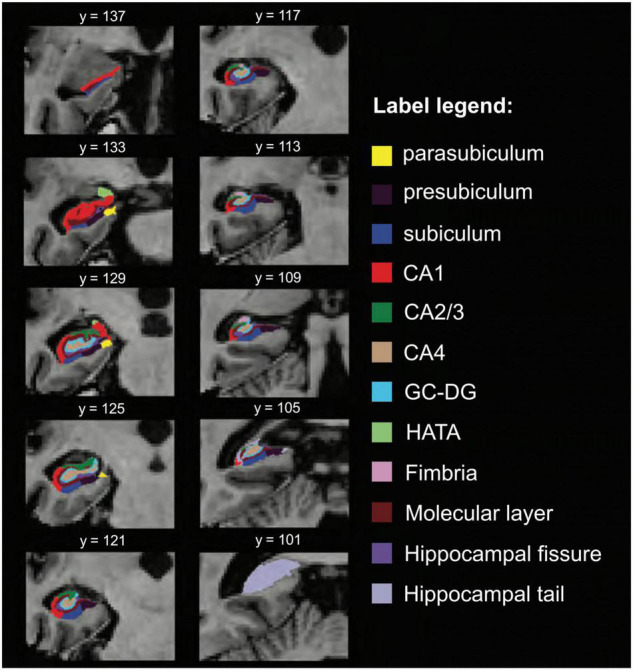
Additional views, hippocampal subfield delineation at 7 Tesla. Coronal view of hippocampal subfield segmentation (radiological display convention) at multiple locations of the same hippocampus. CA, cornu ammonis; GC-DG, granule cell layer of the dentate gyrus; HATA, hippocampus-amygdala-transition-area.

Hippocampal subfield segmentation resulted in accurate delineation of all 12 subfields in left and right hippocampi of all subjects (Figure 1). Accuracy of automatic subfield segmentation obtained by FreeSurfer 6.0 was consistent with hippocampal subfield tissue boundaries, which were visible in the high field strength 7T T1 images. The Anderson-Darling and the Jarque-Bera test for normality confirmed that data for none of the 12 subfields differed significantly from normal distribution (all *p* > 0.1), indicating that data were not skewed by over- or undersegmentation of any hippocampal subregion. Variability was assessed by standard deviation for each subfield. Coronal sections along the entire right-hemispheric hippocampus of one subject are shown in Figure 2.

### Between Group Differences in Hippocampal Subfield Volumes and Cluster Analysis

**FIGURE 3:**
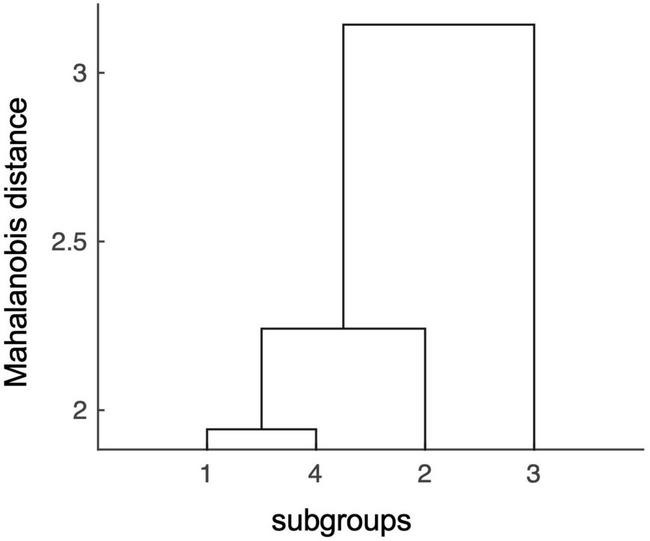
Mahalanobis distances of hippocampal subfield volumes indicate distinct effects conferred by the high-risk subgroup #3. In the Mahalanobis dendrogram statistic independence of data points is indicated by the length of the arms in the hierarchical binary cluster tree. The matrix shows that there is a low degree of statistical relatedness between effects of the high-risk subgroup #3 (high-Aβ-low-episodic memory group), with effects conferred by the other three subgroups: low-Aβ-low-episodic memory (subgroup 1), low-Aβ-high-episodic memory (subgroup 2), and high-Aβ-high-episodic memory (subgroup 4).

The MANOVA with the 12 subfields as dependent variables and the four aforementioned groups as independent variables showed a significant difference in volumes between groups, Wilk’s Lambda = 0.34, *p* = 0.039. The subsequent hierarchical clustering analysis revealed the lowest degree of statistical relatedness of the high-Aβ-low-episodic memory subgroup with the other subgroups, followed by the low-Aβ-high-episodic memory subgroup, while the other two subgroups (low-Aβ-low-episodic memory and high-Aβ-high-episodic memory) were closest related to all others. Cumulative distances (Mahalanobis) for each group were (SD), as defined by MANOVA statistic: Subgroup 1 (low-Aβ-low-episodic memory): 7.4 (1.3); subgroup 4 (high-Aβ-high-episodic memory): 8.6 (1.6); subgroup 2 (low-Aβ-high-episodic memory): 11.8 (2.5); subgroup 3 (high-Aβ-low-episodic memory, *n* = 13): 12.4 (2.5). Figure 3 depicts the distances between subgroups as obtained by Mahalanobis clustering, suggesting subgroup 3 as a separate entity. Based on this finding, we divided the sample into a high- and a low-risk group. The high-risk group corresponded to the high-Aβ-low-episodic memory group (*n* = 13) and the low-risk group (*n* = 48) was comprised of the other three groups. There were no significant differences in age, education and gender distributions between the thus defined high- and low-risk groups (all *p* > 0.05).

### *Post hoc* Differences in Hippocampal Subfield Volumes Between Risk Groups

**FIGURE 4:**
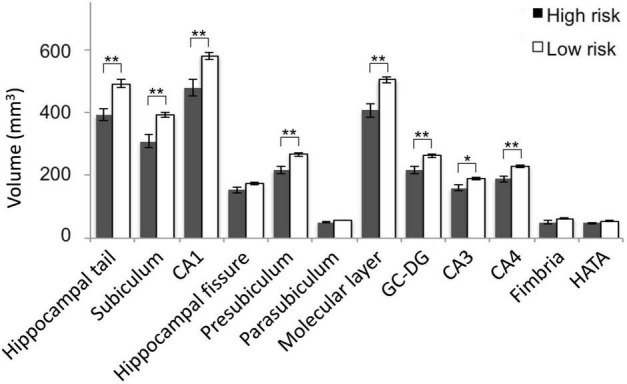
Hippocampal subfield volumes by assumed Alzheimer’s disease (AD) risk. Mean subfield volumes of the high and low risk group with SEM and levels of significance for FDR-corrected *p*-values. SEM, standard error of mean; FDR, false discovery rate; *, FDR-corrected *p* < 0.05; **, FDR-corrected *p* < 0.01. CA, cornu ammonis; GC-DG, granule cell layer of the dentate gyrus; HATA, hippocampus-amygdala-transition-area.

The one-way ANOVAs calculated to examine the differences in volumes of individual subfields between the high and the low-risk group (Figure 4) revealed most significant (both FDR-corrected *p* = 0.002) and strongest effects as indicated by eta squared effect sizes (0.222 and 0.2427, respectively) on subiculum and molecular layer (Table 2). Subiculum and molecular layer were thus further investigated for interactive effects of cortical Aβ and episodic memory performance. The effect sizes and FDR-corrected p-values for all subfields are listed in Table 2. The eta squared effect size for the volume reduction of CA1 (0.2045) was lower than for subiculum and molecular layer as was the level of significance (FDR-corrected *p* = 0.003).

**TABLE 2:** Differences in hippocampal subfield volumes between risk groups.

	Eta squared	df1, df2	*F*(df1,df2)	p	FDR-corrected *p*
Molecular layer	0.2427	1, 58	18.59	<0.001	0.002
Subiculum	0.222	1, 59	16.84	<0.001	0.002
Hippocampal tail	0.21	1, 58	15.42	<0.001	0.003
CA1	0.2045	1, 58	14.91	<0.001	0.003
Presubiculum	0.1893	1, 58	13.55	0.001	0.003
GC-DG	0.1829	1, 59	13.20	0.001	0.003
CA4	0.1806	1, 59	13.01	0.001	0.003
CA3	0.1272	1, 59	8.60	0.005	0.022
Parasubiculum	0.07597	1, 59	4.85	0.032	0.119
Fimbria	0.07559	1, 59	4.82	0.032	0.119
HATA	0.07204	1, 59	4.58	0.036	0.124
Hippocampal fissure	0.06704	1, 58	4.17	0.046	0.142

*CA, Cornu Ammonis; GC-DG, granule cell layer of the dentate gyrus; HATA, hippocampus-amygdala-transition-area.*

### Interaction Between Cortical Amyloid β and Episodic Memory Performance

**FIGURE 5:**
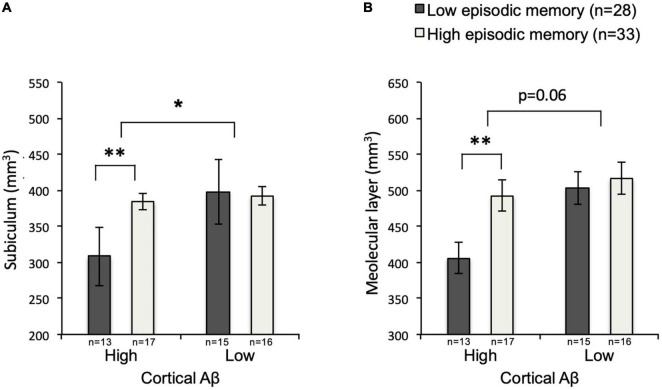
Subiculum and Molecular layer volumes by subgroup. Mean volumes of subiculum **(A)** and molecular layer **(B)** for low-Aβ-high-episodic memory, low-Aβ-low-episodic memory, high-Aβ-high-episodic memory and high-Aβ-low-episodic memory groups with SEM, and level of significance. SEM, standard error of measurement; *p < 0.05; ^**^p < 0.01.

The interaction between cortical Aβ and episodic memory performance was significant for subiculum, *F*(3,57) = 5.90, *p* = 0.018, but not molecular layer, *F*(3,57) = 3.66, *p* = 0.061. Secondary analysis using two-tailed, two-sample (unequal variance) *t*-tests within the high cortical Aβ subgroup revealed lower volumes of the subiculum [*p* = 0.004, mean mm^3^ left + right (SD) high episodic memory: 384.15(47.62); low episodic memory: 308.15(70.21)] and of the molecular layer [*p* = 0.003, mean mm^3^ left + right (SD) high episodic memory: 492.48(60.40); low episodic memory: 406.03(62.74)] in individuals with low versus individuals with high episodic memory performance. This effect was not observable for individuals within the low cortical Aβ subgroup [subiculum, *p* = 0.82, mean mm^3^ left + right (SD) high episodic memory: 392.17(50.52); low episodic memory: 398.27(87.48); molecular layer, *p* = 0.63, mean mm^3^ left + right (SD) high episodic memory: 516.79(71.71); low episodic memory: 502.85(84.34)] (Figure 5).

## Discussion

To our knowledge, this is the first report on an association of AD risk susceptibility, as defined by poorer episodic memory performance within the normal range and amyloid plaque load, with differential hippocampal subfield volume in cognitively normal elderly individuals.

Generally, with today’s clinically available methods, imaging biomarkers for downstream neurodegeneration become positive only after the manifestation of brain amyloidosis in the classical course of AD. Although hippocampal atrophy can be observed up to 10 years before disease onset, the decrease in the total volume of the hippocampus lacks specificity (Tondelli et al., 2012; Hatanpaa et al., 2014; Pini et al., 2016). Advances in structural imaging represent a promising approach for the development of more sensitive and specific methods in this field (Pini et al., 2016).

The mammalian hippocampus consists of several subfields, which have been reported to be variably affected by AD in both post-mortem histopathological and *in vivo* MRI investigations (Rossler et al., 2002; Zheng et al., 2018). Hippocampal subfield volumes might serve as a potential earlier and more specific biomarker for neurodegeneration due to AD (de Flores et al., 2015a; Pini et al., 2016). Histological studies of hippocampal pathology suggest pronounced neuronal loss in the CA1 and subiculum of persons with AD, when compared to healthy individuals (West et al., 1994; Price et al., 2001; Rossler et al., 2002; West et al., 2004).

Numerous *in vivo* MRI hippocampal subfield studies reported a significant volume loss of either almost all subfields with most pronounced effects or focal volume loss of CA1 or CA1 and subiculum in clinical manifest AD (Wang et al., 2006; Frisoni et al., 2008; Mueller and Weiner, 2009; Mueller et al., 2010; Apostolova et al., 2012; Lindberg et al., 2012; Wisse et al., 2014b; de Flores et al., 2015b; Perrotin et al., 2015; Tang et al., 2015; Madusanka et al., 2019). Moreover, subicular volume was found to be the single most predictive hippocampal subfield measure for AD diagnosis (Izzo et al., 2020). The findings of hippocampal subfield atrophy in AD could widely be replicated in patients with MCI, who generally had a more focal pattern of atrophy than AD patients (Chetelat et al., 2008; Mueller and Weiner, 2009; La Joie et al., 2013; de Flores et al., 2015b; Tang et al., 2015; Madusanka et al., 2019; Zhang et al., 2020). Between persons with MCI who converted to AD and non-converters most significant volume differences were reported for the subiculum and presubiculum in the Alzheimer’s Disease Neuroimaging Initiative cohort (Vasta et al., 2016). On the other hand, in MCI, also reports with no relevant atrophy in any hippocampal field have been reported, possibly due to low statistical power (Kerchner et al., 2013; Wisse et al., 2014b). Nevertheless, atrophy of subiculum and CA1 was also detected at very early stages, in cognitively healthy elders, who later developed MCI or AD as well as in elderly subjects with subjective cognitive decline (SCD) (Csernansky et al., 2005; Apostolova et al., 2010; Perrotin et al., 2015). In subjects at risk for AD, defined either by APOE4 carrier status or amyloidosis or positive family history, no conclusive subfield atrophy pattern is established yet (Donix et al., 2010; Dounavi et al., 2020; McKeever et al., 2020; Nadal et al., 2020). In healthy aging, no clear subfield volume evolution could be identified to date. While some studies found CA1 and subiculum to be most affected by age, others found these regions to be relatively preserved (de Flores et al., 2015a; Voineskos et al., 2015; Daugherty et al., 2016; Malykhin et al., 2017; Amaral et al., 2018). In a very recent study neither CA1 nor subiculum volumes expressed significant relationships with age (Bussy et al., 2021). Authors suggest, that the observed discrepancies in findings may be explained by different study designs (Bussy et al., 2021).

In our study we found distinct effects of subgroups as defined by amyloid load and episodic memory performance on hippocampal subfield volumes. Moreover, we observed a hierarchy of statistical independence of the subgroup’s effects ranging from the high-Aβ-low-episodic memory with the highest distance over the low-Aβ-high-episodic memory subgroup to the two other subgroups (low-Aβ-low-episodic memory and high-Aβ-high-episodic memory), that were closest related to all others. This might be consistent with the assumption that the four subgroups represent different stages in the risk spectrum for AD: the high-Aβ-low-episodic memory subgroup might represent the high-risk extreme, while the low-Aβ-high-episodic memory subgroup might be the low-risk extreme, and the low-Aβ-low-episodic memory and high-Aβ-high-episodic memory subgroups might constitute intermediate stages. We feel that this interpretation could be consistent with established concepts of biomarker-based assessment of aging-related brain pathology, where PET and cognitive performance represent independent, non-correlating, but complementary information (Jagust et al., 2009).

Our finding of differential hippocampal volume loss of the subiculum in the AD high-risk group compared to the low-risk group, comprised of the other three subgroups, is in line with the aforementioned results of subiculum atrophy in MCI and AD as well as a reported increased risk for cognitively healthy elders with low subicular volume to develop AD over time (Apostolova et al., 2010; Carlesimo et al., 2015). Moreover, to our knowledge, this is the first study to report a generalized difference in almost all subfields in cognitively normal elders according to AD risk profile, determined by Aβ load and episodic memory performance. While we found no significant difference in chronological age between the investigated groups, variation in Aβ and cognitive performance might reflect differences in biological age.

In contrast to other studies, we found profound but not most significant atrophy in the CA1. Partly, this might be explained by the fact, that atrophy in the CA1 has been suggested to be independent of Aβ plaque accumulation and our study sample was stratified by amyloidosis (La Joie et al., 2013; Ye et al., 2014). Also, it needs to be pointed out, that differing results might on the one hand also be explained by heterogenous methodology regarding hippocampus segmentation. On the other hand, differing results might partly be due to differing MRI resolution in the discussed reports. Except for the studies by Kerchner et al. and Wisse et al. all discussed studies were carried out at MRI field strengths below 7T. Previous MR studies performed at 7T often have investigated small sample sizes, which may limit power for detection of small effects (Kerchner et al., 2010, 2012, 2013, 2014; Boutet et al., 2014; Wisse et al., 2014b). Moreover, results pointing to atrophy in diverging subfields could possibly also be explained by different AD subtypes, that might have differing predilection atrophy sites not only in the neocortex, but also within the hippocampus.

Interestingly, lower subicular volume has already been reported to be associated with poorer performance in a test of immediate and delayed recall of a 15-word list (Carlesimo et al., 2015). Moreover, Zhao et al. (2019) recently reported that volume of left subiculum in comparison to other subfields is most strongly correlated with performance in AVLT measures in a mixed sample of persons with unimpaired cognition, SCD, amnestic MCI or AD. The fact that secondary analysis of our data revealed significant interactive effects of high Aβ and low episodic memory on subicular volume in a group of cognitively unimpaired persons is also in line with a recent report, that found a correlation of subicular volume with amyloid burden and memory decline in a group of subjects with MCI. In this group of MCI subjects, the correlation also existed for molecular layer and CA1 (Zhang et al., 2020). While we found most pronounced effects for the subiculum, the correlations described by Zhang et al. (2020) might represent a later stage of dementia-risk associated hippocampal subfield atrophy.

A major limitation of this study is the fact that it is cross-sectional. While 7T MRI provides very high contrast to noise ratio, longitudinal follow-up is complicated because of frequent changes in hardware and experimental MR-sequence setup. However, as outlined above, there are several other studies published at this point that corroborate subicular volume loss in later stages of AD (La Joie et al., 2013; Vasta et al., 2016; Madusanka et al., 2019; Zhao et al., 2019; Zhang et al., 2020). Another potential limitation lies in the used methodology for hippocampal subfield segmentation. Although the here employed FreeSurfer 6.0 software is the single one most applied software in this context and is increasingly recognized by experts in this field, there is an ongoing debate on the most proper method of segmentation (Wisse et al., 2014a,^2021^; Pini et al., 2016; Samann et al., 2020). The here used version 6.0 has overcome validation concerns of earlier versions with a novel statistical atlas of combined *in vivo* and *ex vivo* data for automatic, Bayesian segmentation (de Flores et al., 2015a; Iglesias et al., 2015). Moreover, there is a debate about the weighing of the source images. While FreeSurfer is able to segment T1 and T2 images as well as their combination, to date there is no clear demonstration of a superiority of either approach (Samann et al., 2020). The here used T1 MP2RAGE images are characterized by a particularly high gray matter/white matter contrast, which is crucial for valid subfield delineation. As longitudinal studies of hippocampal subfields are rare and inconclusive, results from long-term subfield volume observations as well as a harmonization of methods, which is currently undertaken by an international working group, would be of most interest for future studies (Pini et al., 2016).

To discuss another potential limitation of the present study, it has to be pointed out, that a switch in methodology for estimation of Aβ plaque load occurred after 31 subjects were recruited and had received 11C-PiB-PET due to the fact that subjects were recruited from two ongoing studies at our center. Accordingly, the latter 30 subjects were administered the 18F-flutemetamol PET tracer. As this might be a potential limitation, several studies verified the equivalency of 11C-PiB- and 18F-flutemetamol-PET as estimates for cerebral Aβ deposition, though, when both are standardized to cerebellar gray matter, as it was done in the present study (Vandenberghe et al., 2010; Hatashita et al., 2014; Adamczuk et al., 2016).

Taken together our data, as obtained by ultra-high field-strength MRI, indicate that hippocampal subfield atrophy in cognitively normal old-aged adults with higher-than-average cortical Aβ load and low episodic memory performance within the normal range, is a hallmark of increased risk for sporadic AD. The data also suggest that even subtle differences in episodic memory and amyloid load within normal variability can be meaningful as they correspond to significant structural changes as measurable with the sensitive and precise technique of ultra-high field 7TMRI. While our findings are consistent with many studies on brain change in old-aged persons with cognitive impairment, our data may support validity of subicular volume as a surrogate marker for Aβ burden related variation in episodic memory at high age. Thus, subicular atrophy might represent a critical neuroanatomical alteration in the prodrome of AD. Moreover, ultra-high field 7T MRI might be an appropriate technique to help to better identify subjects with a higher biological than chronological age as represented by stratification by biomarkers. Due to its small size, delineation of the entorhinal cortex is difficult even at field strenghts of 7T. In this study we did not suceed at delineating the entorhinal cortex. We hope that future developments in ultra-high field MRI will lead to valid measurement techniques of this region.

While the pathophysiological correlate of lower subicular volume in a context of AD-risk remains unclear at this point, earlier post-mortem studies in AD revealed the subiculum as a hippocampal subfield with particularly high levels of non-heme iron deposition and microglial activation (Zeineh et al., 2015; Madsen et al., 2020). This might accord with the notion of local non-heme iron deposition as a correlate of neurodegenerative brain pathology in AD (van Bergen et al., 2016; van Duijn et al., 2017; Ayton et al., 2019; Zhou et al., 2020). Longitudinal follow-up studies may clarify the temporal sequence of physiological changes, such as aggregation of pathological proteins, local iron deposition, neuronal disintegration, and tissue atrophy. Moreover, the future is believed to lie either in multimodal imaging approaches including Tau- and FDG-PET, fMRI or diffusion-tensor imaging, or even innovative imaging methods allowing for additional information on the underlying pathology that drives hippocampal subfield atrophy (de Flores et al., 2015a).

Last, it has to be mentioned, that these findings of ours also underline the pathogenic relevance of cerebral Aβ burden prior to significant cognitive impairment. Future therapeutic strategies aimed at prevention of AD dementia may allow for subtle Aβ related effects on brain integrity, as measurable by sensitive neuroimaging technology such as 7T MRI.

## Data Availability Statement

The raw data supporting the conclusions of this article will be made available by the authors upon reasonable request after evaluation by the authors and, if applicable, by the local ethics authority, without undue reservation.

## Ethics Statement

The studies involving human participants were reviewed and approved by the cantonal ethics committee of Zurich, Switzerland. The patients/participants provided their written informed consent to participate in this study.

## Author Contributions

SMK: writing of the manuscript, subject recruitment, and statistical data analysis. CS: data processing, statistical analysis, and writing of the manuscript. JMGB: acquisition and processing of MRI data. SJS: acquisition of MRI data and neuropsychological workup of all subjects at time of MRI acquisition. RM and SCS: subject recruitment and extended neuropsychological workup of all participants. KPP and LV: assisted in acquiring data, quality control, and MR-sequence implementation at the 7 Tesla scanner at ETH Zürich. AFG: support of study design and conduct and acquisition of clinical data and PET data. VT and AB: acquisition and analysis of PET data. CH: sponsor of the study. PGU: study design and management, supervision of data processing and statistical analysis, final responsibility, and writing of the manuscript. All authors contributed to the article and approved the submitted version.

## Conflict of Interest

CH is an employee and a shareholder of Neurimmune. The remaining authors declare that the research was conducted in the absence of any commercial or financial relationships that could be construed as a potential conflict of interest.

## Publisher’s Note

All claims expressed in this article are solely those of the authors and do not necessarily represent those of their affiliated organizations, or those of the publisher, the editors and the reviewers. Any product that may be evaluated in this article, or claim that may be made by its manufacturer, is not guaranteed or endorsed by the publisher.

## Footnotes

1
http://surfer.nmr.mgh.harvard.edu/

## Abbreviations

7T: 7 Tesla
A β: amyloid β
AD: Alzheimer’s disease
ANOVA: analysis of variance
AVLT: rey auditory verbal learning test
CA: cornu ammonis
CERAD: consortium to establish a registry for Alzheimer’s disease
GC-DG: granule cell layer of dentate gyrus
GCP: good clinical practice
HATA: hippocampus-amygdala-transition-area
ICV: intracranial volume
MMSE: mini − mental state examination
MANOVA: multiple analysis of variance
MRI: magnetic resonance imaging
PET: positron emission tomography
PiB: pittsburgh compound-B
SCD: subjective cognitive decline
SUVR: standardized uptake value ratio
VLMT: verbal learning and memory test.
